# Effect of maternal nutrition education on early initiation and exclusive breast-feeding practices in south Ethiopia: a cluster randomised control trial

**DOI:** 10.1017/jns.2022.36

**Published:** 2022-05-30

**Authors:** Jatani Admasu, Gudina Egata, Dereje Getahun Bassore, Fentaw Wassie Feleke

**Affiliations:** 1 School of Nutrition, Food Science and Technology, Hawassa University, Hawassa, Ethiopia; 2 Department of Public Health Nutrition and Dietetics, School of Public Health, College of Health Sciences, Addis Ababa University, Addis Ababa, Ethiopia; 3 College of Health Science, Woldia University, Woldia, Ethiopia

**Keywords:** Early initiation of breast-feeding, Exclusive breast-feeding, Nutrition education, Women, ANC, antenatal care, AOR, adjusted odds ratio, BF, breast-feeding, CG, control group, COR, crude odds ratio, CS, caesarean section, CSA, Central Statistical Agency, EBF, exclusive breast-feeding, EIBF, early initiation of breast-feeding, EHNRI, Ethiopian Health and Nutrition Research Institute, FMOH, Federal Ministry of Health, GA, gestational age, HBM, health belief model, HEW, health extension worker, IG, intervention group, IRB, Institutional Review Board, IYCF, infant and young child feeding, PNC, postnatal care, SNNPR, Southern Nations, Nationalities and Peoples Region, SVD, spontaneous vaginal delivery, UNICEF, United Nation International Children's Emergency Fund, WHO, World Health Organization

## Abstract

*Introduction:* Optimal breast-feeding practices make a major contribution to the promotion of healthy growth and development through much prevention of diarrheal and respiratory diseases which majorly cause morbidity and mortality in under-five children. However, breast-feeding practices remain suboptimality in Ethiopia. *Objective:* The study objective was to determine the effect of maternal nutrition education on early initiation and exclusive breast-feeding practice in the Hawela Tulla sub-city. *Methods:* A cluster randomised, parallel-group, single-blinded trial was used. About 310 pregnant women (155 for the intervention group and 155 for the control group) were included. *Result:* An early initiation of breast-feeding was significantly higher among women who received breast-feeding education than those who did not receive (104(72·7 %) *v.* 85(59·9 %), *P* = 0·022) and exclusive breast-feeding practice was also significantly higher among women who received breast-feeding education than those who did not receive (106(74·1 %) *v.* 86(60·6 %), *P* = 0·015). Breast-feeding education [AORs 1·55, 95 % CI (1·02, 2·36)], institutional delivery [AOR 2·29, 95 % CI (1·21, 4·35)], vaginal delivery [AOR 2·85, 95 % CI (1·61, 5·41)] and pre-lacteal feeding [AOR 0·47, 95 % CI (0·25, 0·85)] were predictors of early initiation of breast-feeding. Breast-feeding education [AOR 1·72, 95 % CI (1·12, 2·64)] and institutional delivery [AOR 2·36, 95 % CI (1·28, 4·33)] were also determinants of exclusive breast-feeding practices. *Conclusion:* Breast-feeding education improved early initiation of breast-feeding and exclusive breast-feeding practices. Providing sustained education to women regarding early initiation and exclusive breast-feeding practice should be strengthened.

## Introduction

The World Health Organization (WHO) recommends that all newborn babies should be placed in skin-to-skin contact with their mothers immediately after birth and initiate breast-feeding within 1 h of birth^(1)^. All infants should be exclusively breast-fed for the first 6 months of life and continued breast-feeding for up to 2 years or beyond with timely, adequate, safe and appropriate complementary feeding beginning at 6 months of age^(2)^.

Breast-feeding is universally accepted as the easiest, safest, most effective and most successful intervention for the satisfactory physical and mental health of children and provides lifelong benefits for both the mother and child^(3,4)^. Colostrum, which is thick yellowish breast milk produced during the first days after delivery is highly nutritious and it is the most immunologically protective secretion of the breast during lactose-genesis^(5)^ and serves as antiantibodies for newborn from diseases^(6)^. Breast milk provides the nearly perfect mix of vitamins, protein and fat – everything a baby needs to grow to be ideal nutrition for infants and it is all provided in a more easily digested form^(7)^. It is also lengthening maternal post-partum amenorrhoea and birth interval which is strongly related to infant and young child survival giving women more time to recover from childbirth and baby care^(8)^.

Timely initiation of breast-feeding prevents neonatal early in the first 28 d of birth, including all causes of mortality^(9)^. Children who are exclusively breast-fed in the first 6 months of life are more likely to survive than non-breast-fed children^(6)^ and about 41 % of global under-five deaths that occurs in Sub-Saharan Africa (SSA) are mainly due to inadequate breast-feeding practices combined with a high burden of diseases^(10)^.

Globally, only two out of five (40 %) of newborns are put to the breast within the first hour of life and only 38 % of infants aged 0–6 months are exclusively breast-fed^(2,11)^. A systematic review and meta-analysis studies conducted in Ethiopia indicated that only 61·4 % of newborns are put to the breast within the first hour of birth and only 59·3 % of infants are exclusively breast-fed^(12,13)^. Inadequate information/inadequate knowledge about breast-feeding, place of residence, maternal age, parity, inadequate ANC, place of delivery, caesarean section delivery and PNC affect breast-feeding practices^(14–19)^. Moreover, infant sex, birth weight, pre-lacteal feeding and colostrum feeding also affect breast-feeding practices^(20–22)^.

Breast-feeding is the best way of providing ideal food for infants. However, many mothers are unable to practice early initiation of breast-feeding and exclusive breast-feeding as recommended. Suboptimal breast-feeding practices increase the risk of nutritional imbalances and infectious diseases, which in turn hinder the physical growth, and health of the infant and it imposes lifelong impacts like poor school performance, reduced productivity and impaired intellectual development^(10,23,24)^.

Worldwide, 823 000 children under 5 years of age are suffering from improper breast-feeding practice including pre-lacteal feeding^(25)^. This practice contributes to 45 % of neonatal mortality, 11·6 % of under-five mortalities, 30 % of diarrheal mortality and 18 % of acute respiratory deaths among infants^(25)^. Only 61·4 % of newborns are breast-fed within the first hour of life, 25·29 % of children are given prolateral foods, and only 58 % of children under the age of 6 months are exclusively breast-fed^(12,25,26)^.

A previously done study in Hawela Tulla sub-city of Hawassa town showed that understanding the benefits of proper breast-feeding practices remains a significant gap in the community in which only 37 % of women have good knowledge about breast-feeding practices^(27)^. Another study done in the Hula District of Sidama Zone showed a significant gap in breast-feeding practices in which only nearly half (50·6 %) of mothers initiate breast-feeding early, 25·5 % of women give pre-lacteal feeds to their newborns and only 43·1 % of the mothers practice optimal breast-feeding^(28)^. The only limited study is found regarding the effect of nutrition education on breast-feeding practices in Ethiopia^(15)^. According to the study, prenatal maternal education does not improve the early initiation of breast-feeding significantly, but it significantly improved exclusive breast-feeding the at third month post-partum. Hence, the present study evaluated the effect of breast-feeding education in improving the early initiation of breast-feeding and exclusive breast-feeding practices.

## Methods

### Study area

Hawassa city is the capital of the Southern Nations, Nationalities and Peoples Region (SNNPR), and the Sidama zone is located 273 km south of Addis Ababa, the capital of Ethiopia. It is located at 70′03″ latitude and 80′29″ east longitude. The city is divided into 8 sub-cities and 32 kebeles. According to the report of the housing and population census, the projected population of Hawassa city Administration in 2011 was 374 034 out of which 183 277 were females. The annual population growth rate is 4·02 %^(29)^. The study was conducted in Hawela Tulla sub-city which is a rural sub-city and the largest sub-city in terms of area. The sub-city has one primary hospital, five governmental health centres and one private health centre.

### Study period and study design

The study was conducted from June 2019 to February 2020 with a cluster randomised, parallel-group, single-blinded trial study design.

### Population

All pregnant women whose gestational age was 26–32 weeks and who resided in Hawela Tulla sub-city of Hawassa town were the source population. All randomly selected pregnant women whose gestational age was 26–32 weeks in selected kebeles of Hawela Tulla sub-city of Hawassa town were the study population.

### Inclusion and exclusion criteria

Pregnant women whose gestational age was 26–32 weeks and permanent residents of the study area were included in the study. Pregnant women who were critically ill or unable to communicate during the study period were excluded from the study.

### Sample size determination

The sample size was determined using the formula for the difference between two population proportions with the following assumptions: 5 % level of significance, 80 % power, for the first specific objective, taking 96 and 84 % prevalence of early initiation of breast-feeding in the intervention group and in the control group, respectively, from a study done in Pakistan^(30)^. The calculated sample size was 188 (94 for each group) and after using a design effect of 1·5 and adding 10 % lost to follow-up, the final sample size was 310 (155 for each group). For the second specific objective, taking 85·5 and 64 % prevalence of exclusive breast-feeding in the intervention group and in the control group, respectively, from a study done in India^(31)^. The calculated sample size was 120 (60 for each group) and after using a design effect of 1·5 and adding 10 % lost to follow-up, the final sample size was 198 (99 for each group).

The sample size was calculated using formula
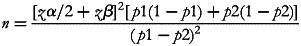
where *n* is the number of pregnant women required, *p*1 is the proportion in the intervention group, *p*2 is the proportion in the control group, *zα*/2 is 1·96 for 95 % confidence level and *zβ* is 0·84 for 80 % power.

The sample size for the third and fourth objectives was calculated using G* power version 3.1.9.2 software assuming two groups, using alpha 0·05, power 0·8, effect size 0·3 and allocation ratio 1:1. The calculated sample size was 282 (141 for each group). The calculated largest final sample size was 310 (155 for each group).

### Sampling technique and procedure

Hawela Tulla sub-city was purposely selected. From a total of 12 kebeles in the sub-city, kebeles were selected randomly and were assigned into the intervention and control groups. Then, the proportion to size allocation was done to select the required number of samples from each selected kebeles. Sample size was proportionally allocated using the formula: *ni = n*/*N*, where *ni* is the required number of pregnant women, *n* is the total sample size and *N* is the total number of pregnant women. *n*/*N* is the sampling fraction. *n* = 310 (155 for intervention and 155 for control groups).

**Fig. 1.**
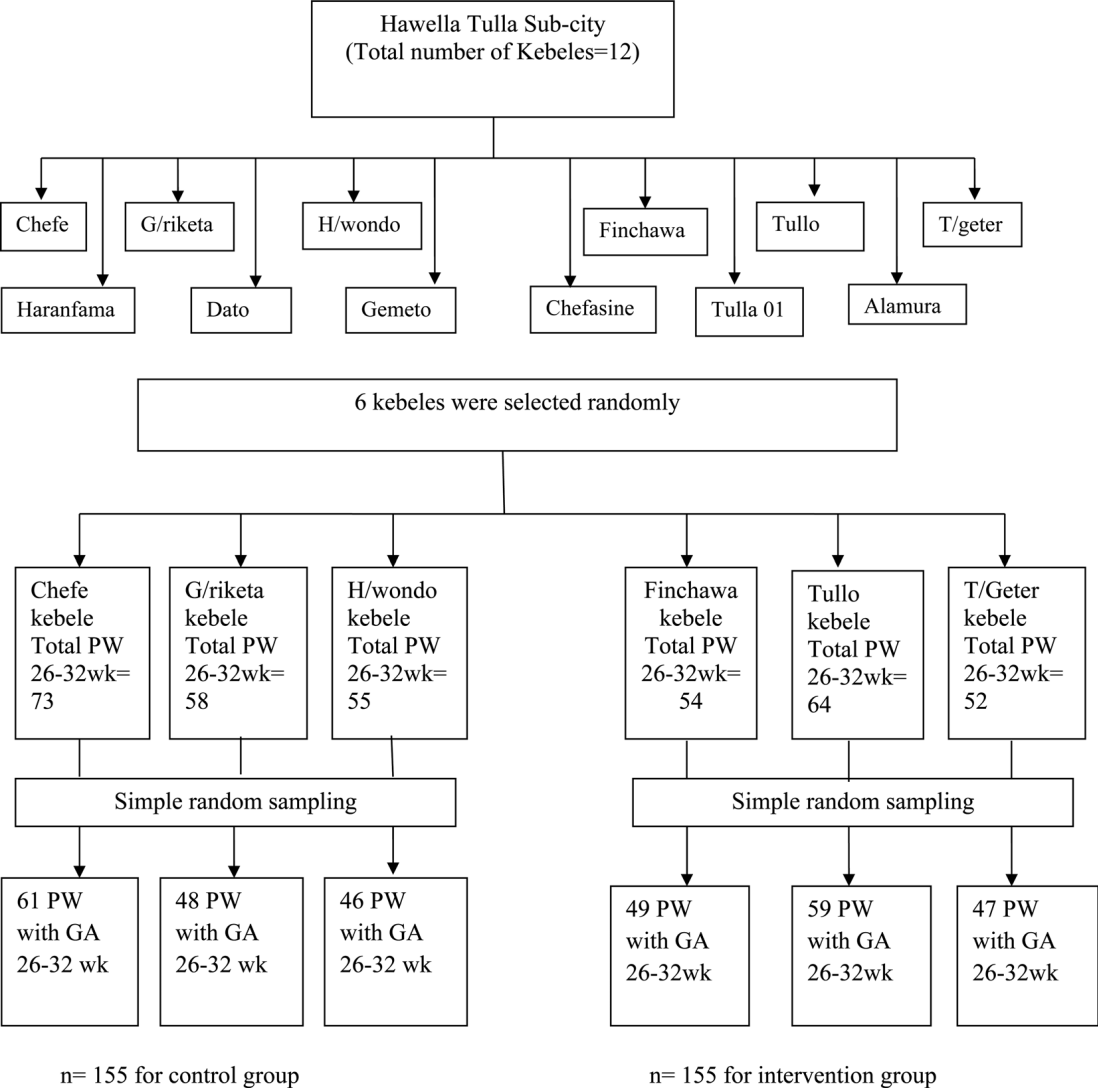
Sampling procedure for effect of maternal education on breast-feeding practices in Hawela Tulla sub-city of Hawassa 2020.

*ni* = *Ni*/N*n. Then by using this formula pregnant women with gestational age of 26–32 weeks were selected from each kebele using simple random sampling (Fig. 1).

## Definition of terms

**Colostrum:** The first milk produced by the mammary glands^(32)^.

**Pre-lacteal feeding:** Giving any solid or liquid foods other than breast milk during the first 3 d after birth^(25)^.

**Early initiation of breast-feeding:** Putting the newborn to the breast within 1 h of birth^(18)^.

**Exclusive breast-feeding:** The infant receives only breast milk and no additional food, water or other liquids (with the exception of medicine and vitamins, if needed) up to 6 months of age^(11)^.

**Optimal breast-feeding:** Breast-feeding be initiated within 1 h of birth, that it continues with no other foods or liquids for the first 6 months of life, and be continued with complementary feeding (breast-feeding with other age-appropriate foods) until at least 24 months of age^(1,6)^.

**Parity:** The number of times that a woman has given birth to a fetus with a gestational age of 24 weeks or more, regardless of whether the child was born alive or was stillborn.

**Gravidity:** The number of times that a woman has been pregnant.

**Control group:** The group who not received nutrition education.

**Intervention group:** The group who received nutrition education.

**Nutrition education:** The process of teaching the science of nutrition to an individual or group.

In the present study, it was given to a group of study participants by dividing the education into small sessions so that the message could be absorbed and used by them.

## Data collection

### Data collection instrument

Baseline and end-line data were collected using a structured questionnaire by face-to-face interviews. The questionnaire for socio-demographic and economic characteristics was developed by adopting tools from the Ethiopian demographic health survey^(26)^ and the questionnaire for breast-feeding practices was developed by adopting tools from WHO Indicators for assessing infant and young child feeding practices^(3,33)^ and from different studies^(34)^. The questionnaire was prepared in English language and translated into the Amharic language.

### Data collectors

Six health professionals (nurses) who can speak the local language (Sidamo ago) were recruited for data collection and two health officers were recruited as supervisors. The data collectors and supervisors were trained before starting of the data collection.

### Nutrition education process

#### Health belief model

Health belief model (HBM) is the most commonly used theory to change health behaviours. According to the model, the messages will achieve optimal behaviour change if they successfully target perceived barriers, benefits, self-efficacy and threat^(35)^. It is based on the belief that the perception an individual has determined their success in taking on that behaviour change.

Individual perception about health behaviour is controlled by modifying variables, cues to action and self-efficacy, and also a successful promotion of health behaviour depends on the understanding of the factors that influence perception. Women will breast-feed as recommended if they are influenced to develop a positive perception about breast-feeding^(36)^. Positive perception and intention towards health behaviour will result in self-efficacy and intention to promote a health behaviour^(36,37)^. Studies done using health belief model brought a significant change regarding maternal breast-feeding knowledge and attitude, self-efficacy and perceived barriers. According to a study done in Greece, women in the intervention group had a more positive attitude towards breast-feeding (73·5 % *v.* 66·1 %), greater knowledge (14·6 % *v.* 13·1 %) and more breast-feeding self-efficacy (51·4 % *v.* 45·6 %) compared to the control group. Furthermore, they had significantly less perceived barriers regarding breast-feeding (27·4 % *v*. 31·0 %)^(38)^.

#### Nutrition education

After collecting the baseline data, nutrition education using lectures was given once every week for 3 weeks to the women in the intervention group using the local language, poster, manual and discussion on topics relevant to the study. Additionally, one session of education was given to the women in the intervention group at third post-partum month. A health professional (BSc Nurse) who can speak the local language was selected to give nutrition education and was given detailed information on the content of each session. Each session lasted for 30–35 min. After imparting nutrition education, questionnaire was used to assess and compare the breast-feeding practice between the intervention and control groups. After collecting end-line data, nutrition education was also given for the women in the control group for ethical purpose.

#### Nutrition education lesson

Baseline data were taken both from the control and interventional group before the starting of the intervention. Appropriate time and place were fixed for the education in consultation with health extension workers. Nutrition education was given based on Infant & Young Child Feeding. Quick Reference Book (0–24 months) prepared by Federal Ministry of Health of Ethiopia and Alive & Thrive-Ethiopia^(39)^ and also from findings from different published researches^(28,40,41)^.

The following topics were addressed during the education of mothers after they were introduced to the objectives of the research and have given their consent.

##### Lesson I

What proper breast-feeding practice is (including the time of breast-feeding initiation, colostrum feeding and avoidance of any pre-lacteal feeds, frequency of breast-feeding and switching from one breast to the other during suckling, exclusive breast-feeding and continuation of breast-feeding).

##### Lesson II

Health and nutritional benefits of proper breast-feeding practice both to the infant and the mother (including benefits of early initiation of breast-feeding both to the infant and the mother, benefits of feeding colostrum and not giving pre-lacteal feeds, benefits of exclusive breast-feeding and continuation of breast-feeding).

##### Lesson III

Harms of inappropriate breast-feeding practices (including harms of delaying initiation of breast-feeding, infrequent breast-feeding and switching from one breast to the other before finishing the first one, discarding (not feeding) colostrum, giving prolateral feeds, non-exclusive breast-feeding and early introduction of complementary foods). Common factors that can affect early initiation an exclusive breast-feeding practices (including common wrong perceptions, social and cultural issues that affect early initiation of breast-feeding, colostrum feeding, pre-lacteal feeding and exclusive breast-feeding practices). Self-confidence/efficacy to practice early initiation of breast-feeding and exclusive breast-feeding.

##### Lesson IV

Duration of exclusive breast-feeding, appropriate time of introduction of complementary foods and continuation of breast-feeding up to 2 years. Benefits of exclusive breast-feeding and continuation of breast-feeding both to the infant and the mother. Harms of non-exclusive breast-feeding and early introduction of complementary foods. Self-confidence/efficacy to practice exclusive breast-feeding. The lesson was supported by a poster demonstration and it was carried out till the women were able to understand the conversation which was confirmed by check questions.

The women in the control group were not exposed to the details of education prepared. Women in the control group received the routine care offered by the HEWs and WDA leaders working in their cluster, similar to that received by women in the intervention group^(42)^. The current Ethiopian standard/routine prenatal and postnatal care by HEWs includes providing four focused prenatal visits, developing an individualised birth preparedness and complication readiness plan, accompanying a woman to a health facility during delivery, and conducting four postnatal visits^(43,44)^. Moreover, as part of the community-based nutrition programme, HEWs are expected to deliver the following key breast-feeding and nutrition messages to mothers during the monthly growth monitoring sessions or during antenatal or postnatal care visits: the importance of antenatal care, maternal nutrition during pregnancy, and breast-feeding, early initiation of breast-feeding, proper positioning and attachment, EBF for 6 months, breast-feeding on demand and complementary feeding^(45)^. WDA leaders also support the HEWs by educating and mobilising communities to use key available health services, including dissemination of essential health messages such as infant and young child feeding practices.

### Data quality control measures

Data collectors and supervisors were trained for 2 d before starting data collection. The training focused on time management, communication skills, appropriate questionnaire filling and the way of supervision. The questionnaire was pre-tested on 5 % of the calculated final sample size population in Gemeto kebele of Hawella Tula Sub-city. Cross checking of all questionnaires was done to ensure the completeness of the data and it was cleaned and coded before entry.

### Data processing and analysis

The collected data were checked manually for completeness, coded and entered using Statistical Package for Social Science (SPSS) version 20 software. Analysis was done using SPSS version 20 and STATA version 14.0 software. The normality of the data was checked using the Kolmogorov–Smirnov and Shapiro–Wilk tests. Hausman's test was done to choose between conditional random-effect and fixed-effect logistic regression and it was found significant. So, fixed-effect conditional logistic regression was done to identify determinants of breast-feeding practices. Pearson's *χ*^2^ test was used to see the difference in socio-demographic and economic variables and also to compare the knowledge and practice of breast-feeding between the two groups. The household wealth index was computed using principal component analysis. Frequency distribution, measures of central tendency and desperation were analysed to describe the result. Bivariable and multivariable conditional fixed-effect logistic regression analyses were done. Variables with a *P*-value less than 0·25 were considered for multivariable analysis to control for all possible confounders and to identify determinants of breast-feeding practices. The level of statistical significance was declared at *P*-value less than 0·05.

## Result

About 310 pregnant women enrolled during the starting of the study. Of these, 155 were in the intervention group and another 155 were in the control group. Data were summarised at the baseline for 310 participants and at the end-line for 285 participants who went through the whole set of education, follow-up and data collection. Eleven participants from the intervention group (four women from Finchawa, four women from Tullo and three women from Tulla Geter kebeles), and thirteen participants from the control group (five women from Chefe, five women from G/riketa and three women from H/wondo kebeles) did not complete the study and were not included during the end-line data collection since they were lost to follow-ups. The lost to follow-up percentage was 7·74 % (7·09 % in intervention and 8·39 % in control groups). Additionally, one of the study participants from Finchawa kebele has given birth to twin infants and she was excluded from the study.

### Socio-demographic characteristics of study participants

The mean (±sd) age in years in intervention and control groups was 25·08 (±4·11) and 24·95 (±3·81), respectively, having no statistically significant difference across the groups. Almost all 309(99·7 %) study participants were married. Among the women assigned to intervention and control groups, 111(35·8 %) of them were unable to read and write while 109(35·3 %) of partners of the respondents attended primary school. The majority of study participants 140(90·3 %) in intervention and 148(95·5 %) in control groups were house wives. Regarding the wealth index, 116(37·4 %) of them were in the middle category (Table 1). More than half 187(60·3 %) of the study participants had 1–2 children. The mean (± sd) parity in intervention and control groups was 1·59 (±1·194) and 1·56 (±1·129), respectively, having no statistically significant difference across the groups (Table 1).

**Table 1.** Socio-demographic characteristics of the women in intervention and control groups in Hawela Tulla sub-city of Hawassa town, in 2020 (*n* 310)

Variables	Intervention group	Control group	Total	*P*-value
Age category in years (*n* 310)				0·799
15–19	13(8·4 %)	11(7·1 %)	24(7·7 %)	
20–24	57(36·8 %)	60(38·7 %)	117(37·7 %)	
25–29	63(40·6 %)	67(43·2 %)	130(42·0 %)	
≥30	22(14·2 %)	17(11·0 %)	39(12·6 %)	
Mean (±sd) age	25·08 (±4·11)	24·95 (±3·81)	25·02 (±3·96)	
Marital status (*n* 310)				0·317
Married	155(100 %)	154(99·4 %)	309(99·7 %)	
Divorced		1(0·6 %)	1(0·3 %)	
Maternal educational status (*n* 310)				0·669
Cannot read and write	53(34·2 %)	58(37·4 %)	111(35·8 %)	
Can read and write	38(24·5 %)	30(19·4 %)	68(21·9 %)	
Primary (1–8)	50(32·3 %)	55(35·5 %)	105(33·9 %)	
Secondary (9–12) and above	14(9 %)	12(7·7 %)	26(8·4 %)	
Partner's educational status (*n* 309)				0·256
Cannot read and write	38(24·5 %)	44(28·6 %)	82(26·5 %)	
Can read and write	26(16·8 %)	24(15·6 %)	50(16·2 %)	
Primary (1–8)	62(40·0 %)	47(30·5 %)	109(35·3 %)	
Secondary (9–12) and above	29(18·7 %)	39(25·3 %)	68(22·0 %)	
Mother's occupation (*n* 310)				0·344
House wife	140(90·3 %)	148(95·5 %)	288(92·9 %)	
Merchant	8(5·2 %)	3(1·9 %)	11(3·5 %)	
Government employee	5(3·2 %)	3(1·9 %)	8(2·6 %)	
Others (daily labourer, NGO employee)	2(1·3 %)	1(0·7 %)	3(1·0 %)	
Wealth index (*n* 310)				0·927
Richest	8(5·2 %)	6(3·9 %)	14(4·5 %)	
Richer	20(12·9 %)	24(15·5 %)	44(14·2 %)	
Middle	59(38·1 %)	57(36·7 %)	116(37·4 %)	
Poorer	57(36·8 %)	55(35·5 %)	112(36·1 %)	
Poorest	11(7·0 %)	13(8·4 %)	24(7·8 %)	
Parity (*n* 310)				0·440
Primipara	34(21·9 %)	29(18·7 %)	63(20·3 %)	
1–2	88(56·8 %)	99(63·9 %)	187(60·3 %)	
≥3	±33(21·3 %)	27(17·4 %)	60(19·4 %)	
Mean (±sd) parity	1·59 (±1·194)	1·56 (± 1·129)	1·58 (± 1·160)	
Gestational age (*n* 310)				0·986
Mean (±sd) gestational age	28·88(± 2·017)	29·10(± 2·012)	28·99(± 2·014)	

The *P*-value is obtained from *χ*^2^ test.

### Knowledge of the study participants about breast-feeding

Prior to receiving nutrition education, 131(84·5 %) in intervention and 135(87·1 %) in control groups heard information about breast-feeding. Among the study participants who heard information about breast-feeding, 119(90·8 %) in intervention and 125(92·6 %) in control groups heard the information from health professionals. From the study participants, 118(76·1 %) in intervention and 121(78·1 %) in control groups knew the benefits of breast-feeding to the infant, but 103(66·5 %) in intervention and 95(61·3 %) in control groups were not aware of the benefits of breast-feeding gives to the mother. Among the study participants, 62(40 %) in intervention and 58(37·4 %) in control groups were not aware of the WHO recommendation on the time of breast-feeding initiation, and also 51(32·9 %) in intervention and 48(31·0 %) in control groups were not aware of the duration of exclusive breast-feeding of the WHO recommendation (Table 2).

**Table 2.** Knowledge of women prior to receiving education in intervention and control groups in Hawela Tulla sub-city of Hawassa town, in 2020 (*n* 310)

Variables	Intervention group	Control group	Total	*P*-value
Received information on breast-feeding (*n* 310)				0·515
Yes	131(84·5 %)	135(87·1 %)	266(85·8 %)	
No	24(15·5 %)	20(12·9 %)	44(14·2 %)	
Source of information about breast-feeding (*n* 266)^a^				
Health professionals	119(90·8 %)	125(92·6 %)	244(91·7 %)	0·604
Family/relatives/friends	12(9·2 %)	8(5·9 %)	20(7·5 %)	0·317
Media (magazines, newspapers, TV, radio)	6(4·6 %)	5(3·7 %)	11(4·1 %)	0·720
Breast-feeding gives benefit to the infant (*n* 310)				0·685
Yes	118(76·1 %)	121(78·1 %)	239(77·1 %)	
No	37(23·9 %)	34(21·9 %)	71(22·9 %)	
Benefits of BF to the infant (*n* 239)^a^				
Provide perfect nutrition	89(75·4 %)	89(73·6 %)	178(74·5 %)	0·740
Prevents disease	53(44·9 %)	60(49·6 %)	113(47·3 %)	0·470
Promotes growth and development	48(40·7 %)	60(49·6 %)	108(45·2 %)	0·166
Promotes baby–mother bonding	23(19·5 %)	34(28·1 %)	57(23·8 %)	0·118
Breast-feeding gives benefit to the mother (*n* 310)				0·344
Yes	52(33·5 %)	60(38·7 %)	112(36·1 %)	
No	103(66·5 %)	95(61·3 %)	198(63·9 %)	
Benefits of BF to the mother (*n* 112)^a^				
Uterine contraction	19(36·5 %)	22(36·7 %)	41(36·6 %)	0·989
Helps in placental expulsion	15(28·8 %)	22(36·7 %)	37(33·0 %)	0·380
Protects from blood loss	14(26·9 %)	15(25·0 %)	29(25·9 %)	0·817
Delays pregnancy	18(34·6 %)	24(40·0 %)	42(37·5 %)	0·557
Prevents maternal breast problems	19(36·5 %)	28(46·7 %)	47(42·0 %)	0·279
Awareness about time of breast-feeding initiation (*n* 310)				
Within 1 h	93(60·0 %)	97(62·6 %)	190(61·3 %)	0·727
After 1 h	62(40 %)	58(37·4 %)	120(38·7 %)	
Awareness about duration of exclusive breast-feeding(*n* 310)				0·808
6 months	104(67·1 %)	107(69·0 %)	211(68·1 %)	
Other than 6 months	51(32·9 %)	48(31·0 %)	99(31·9 %)	
Pre-lacteal should be given (*n* 310)				0·897
Yes	40(25·8 %)	41(26·5 %)	81(26·2 %)	
No	115(74·2 %)	114(73·5 %)	229(73·8 %)	
Colostrum should be given (*n* 310)				0·555
Yes	125(80·6 %)	129(83·2 %)	254(81·9 %)	
No	30(19·4 %)	26(16·8 %)	56(18·1 %)	

a The percent exceeds 100 due to multiple response questions.

### Obstetric characteristics of study participants

The majority of the study participants 187(65·6 %) had less than 4 antenatal care follow-ups. The mean (±sd) number of antenatal care attendance of the study participants was 3·01 (±0·884) and 3·11 (±0·787) in intervention and control groups, respectively. More than half 199(69·8 %) of the study participants had given birth in health facilities while the rest 86(30·2 %) of them had given birth at home and more than three-fourth 225(78·9 %) of the women had given birth vaginally while the rest 60(21·1 %) had given birth through caesarean section. More than three quarter 228(80·0 %) of the women had gained postnatal care after giving birth and 147(51·6 %) of the infants are females. There is no statistically significant difference regarding number of antenatal follow-ups, place of delivery, mode of delivery and postnatal care between the intervention and control groups (Table 3).

**Table 3.** Obstetric characteristics of women in intervention and control groups in selected kebeles of Hawela Tulla sub-city of Hawassa town, in 2020 (*n* 285)

Variables	Intervention group (*n* 143)	Control group (*n* 142)	Total (*n* 285)	*P*-value
Number of ANC visits (*n* 285)				0·770
1–3	95(66·4 %)	92(64·8 %)	187(65·6 %)	
≥4	48(33·6 %)	50(35·2 %)	98(34·4 %)	
Mean (±sd) ANC no	3·01 (±0·884)	3·11 (±0·787)	3·06 (±0·837)	
Place of delivery (*n* 285)				0·766
Health institution	101(70·6 %)	98(69·0 %)	199(69·8 %)	
Home	42(29·4 %)	44(31·0 %)	86(30·2 %)	
Delivery mode (*n* 285)				0·582
SVD	111(77·6 %)	114(80·3 %)	225(78·9 %)	
C/S	32(22·4 %)	28(19·7 %)	60(21·1 %)	
Sex of infant (*n* 285)				0·857
Male	70(49·0 %)	68(47·9 %)	138(48·4 %)	
Female	73(51·0 %)	74(52·1 %)	147(51·6 %)	

The *P*-value is obtained from *χ*^2^ test.

### Breast-feeding practices of study participants

The higher proportion of women who received education intervention have initiated breast-feeding early than those who did not receive being 104[72·7 %, 95 % CI (64·7 %, 79·8 %)] *v.* 85[59·9 %, 95 % CI (51·3 %, 67·9 %)] having a statistically significant difference between the groups (*P* = 0·022). Exclusive breast-feeding practice was also higher among women who received education intervention than those who did not receive being 106[74·1 %, 95 % CI (66·1 %, 81·1 %)] *v.* 86[60·6 %, 95 % CI (52·0 %, 68·7 %)] having a statistically significant difference (*P* = 0·015) across the groups. About 24(16·8 %) of women in the intervention group, and more than a quarter 41(28·9 %) of women in the control group have given pre-lacteal feeds to their infants with a statistically significant difference across the groups (*P* = 0·015), and the majority of study participants (130(90·9 %) in intervention and 122(85·9 %) in control groups) have given colostrum to their infants (Table 4).

**Table 4.** Breast-feeding practices of women in intervention and control groups in selected kebeles of Hawela Tulla sub-city of Hawassa town, in 2019/2020 (*n* 285)

Variables	Intervention group (*n* 143)	Control group (*n* 142)	Total (*n* 285)	*P*-value
Breast-feeding initiation (*n* 285)				0·022
Early	104(72·7 %)	85(59·9 %)	189(66·3 %)	
Delayed	39(27·3 %)	57(40·1 %)	96(33·7 %)	
Exclusive breast-feeding (*n* 285)				0·015
Yes	106(74·1 %)	86(60·6 %)	192(67·4 %)	
No	37(25·9 %)	56(39·4 %)	93(32·6 %)	
Pre-lacteal given (*n* 285)				0·015
No	119(83·2 %)	101(71·1 %)	220(77·2 %)	
Yes	24(16·8 %)	41(28·9 %)	65(22·8 %)	
Colostrum given (*n* 285)				0·188
Yes	130(90·9 %)	122(85·9 %)	252(88·4 %)	
No	13(9·1 %)	20(14·1 %)	33(11·6 %)	
Postnatal care (*n* 285)				0·441
Yes	117(81·8 %)	111(78·2 %)	228(80·0 %)	
No	26(18·2 %)	31(21·8 %)	57(20·0 %)	

The *P*-value is obtained from *χ*^2^ test.

### Determinants of early initiation of breast-feeding

Thirteen factors (receiving education intervention, maternal age, maternal educational status, partner's educational status, wealth index, parity, number of antenatal cares, place of delivery, mode of delivery, sex of infant, pre-lacteal feeding, colostrum feeding and postnatal care) were included in the bivariable conditional fixed-effect logistic regression. In bivariable conditional fixed-effect logistic regression, receiving education intervention, place of delivery, mode of delivery, pre-lacteal feeding, colostrum feeding and gaining postnatal care were significantly associated with early initiation of breast-feeding. In multivariable conditional fixed-effect logistic regression, receiving education intervention, place of delivery, mode of delivery and prolateral feeding were significantly associated with early initiation of breast-feeding. Women who received education intervention were 1·55 times more likely [AOR 1·55, 95 % CI (1·02, 2·36)] to initiate breast-feeding earlier as compared with those who did not gain. Women who gave birth at health facilities were 2·29 times more likely [AOR 2·29, 95 % CI (1·21, 4·35)] to initiate breast-feeding earlier compared with women who gave birth at home and also those who gave birth vaginally were 2·85 times more likely [AOR 2·85, 95 % CI (1·61, 5·41)] to initiate breast-feeding earlier than those who gave birth by caesarean section. Furthermore, study participants who gave pre-lacteal feeds were 53 % less likely [AOR 0·47, 95 % CI (0·25, 0·85)] to initiate breast-feeding early compared with those who did not give pre-lacteal feeds (Table 5).

**Table 5.** Determinants of EIBF among women in Hawela Tulla sub-city of Hawassa town, in 2020 (*n* 285)

Variables	Total no	EIBF		COR (95 % CI)	AOR (95 % CI)
		Yes	No		
Intervention					
Yes	143	104(72·7 %)	39(27·3 %)	1·640(1·091, 2·466)*	1·549(1·015, 2·364)**
No	142	85(59·9 %)	57(40·1 %)	1	1
Age					
15–19	23	13(56·5 %)	10(43·5 %)	0·757(0·377, 1·523)	1·175(0·489, 2·825)
20–24	105	67(63·8 %)	38(36·2 %)	0·719(0·360, 1·439)	1·484(0·539, 4·087)
25–29	119	79(66·4 %)	40(33·6 %)	0·504(0·205, 1·241)	1·474(0·359, 6·048)
≥30	38	26(68·4 %)	12(31·6 %)	1	1
Maternal educational status					
Cannot read and write	103	66(64·1 %)	37(35·9 %)	0·936(0·542, 1·617)	1·252(0·564, 2·778)
Can read and write	62	40(64·5 %)	22(35·5 %)	1·060(0·666, 1·688)	1·316(0·569, 3·044)
Primary (1–8)	96	60(62·5 %)	36(37·5 %)	0·541(0·212, 1·378)	0·900(0·262, 3·088)
Secondary (9–12) and above	24	19(79·2 %)	5(20·8 %)	1	1
Partner's educational status					
Cannot read and write	76	47(61·8 %)	29(38·2 %)	0·825(0·434, 1·567)	0·762(0·328, 1·772)
Can read and write	45	31(68·9 %)	14(31·1 %)	0·950(0·580, 1·556)	0·757(0·318, 1·805)
Primary (1–8)	101	61(60·4 %)	40(39·6 %)	0·738(0·408, 1·335)	0·675(0·233, 1·962)
Secondary (9–12) and above	63	46(73·0 %)	17(27·0 %)	1	1
Wealth index					
Poorest	22	12(54·5 %)	10(45·5 %)	0·801(0·400, 1·605)	1·064(0·464, 2·442)
Poorer	102	61(59·8 %)	41(40·2 %)	0·637(0·315, 1·290)	1·009(0·412, 2·474)
Middle	106	72(67·9 %)	34(32·1 %)	0·488(0·203, 1·172)	1·059(0·327, 3·431)
Richer	41	30(73·2 %)	11(26·8 %)	0·421(0·116, 1·531)	0·867(0·154, 4·881)
Richest	14	10(71·4 %)	4(28·6 %)	1	1
Parity					
Primipara	61	36(59·0 %)	25(41·0 %)	1	1
1–2	164	106(64·6 %)	58(35·4 %)	1·929(0·982, 3·790)	1·949(0·673, 5·642)
≥3	60	43(71·7 %)	17(28·3 %)	1·690(0·927, 3·081)	1·488(0·656, 3·376)
Number of ANC visits					
1–3	187	116(62·0 %)	71(38·0 %)	1	1
≥4	98	69(70·4 %)	29(29·6 %)	0·884(0·577, 1·357)	1·125(0·707, 1·790)
Place of delivery					
Health institution	198	147(74·2 %)	51(25·8 %)	2·147(1·436, 3·280)*	2·291(1·209, 4·340)**
Home	87	38(43·7 %)	49(56·3 %)	1	1
Delivery mode					
SVD	225	151(67·1 %)	74(32·9 %)	1·566(1·003, 2·444)*	2·851(1·611, 5·407)**
C/S	60	34(56·7 %)	26(43·3 %)	1	1
Sex of infant					
Male	138	90(65·2 %)	48(34·8 %)	1·084(0·726, 1·618)	1·261(0·827, 1·921)
Female	147	95(64·6 %)	52(35·4 %)	1	1
Pre-lacteal given					
No	220	161(73·2 %)	59(26·8 %)	1	1
Yes	65	24(36·9 %)	41(63·1 %)	0·368(0·244, 0·555)*	0·465(0·254, 0·853)**
Colostrum given					
Yes	252	172(68·3 %)	80(31·7 %)	1	1
No	33	13(39·4 %)	20(60·6 %)	0·503(0·301, 0·840)*	0·711(0·362, 1·393)
Postnatal care					
Yes	228	163(71·5 %)	65(28·5 %)	2·156(1·404, 3·309)*	1·340(0·806, 2·228)
No	57	26(45·6 %)	31(54·4 %)	1	1

* *P*-value < 0.25.

** *P*-value < 0.05.

### Determinants of exclusive breast-feeding practices

Thirteen factors (receiving education intervention, maternal age, maternal education status, partner's education status, wealth index, parity, number of ANC visits, place of delivery, mode of delivery, sex of infant, pre-lacteal feeding, colostrum feeding and postnatal care) were included in the bivariable conditional fixed-effect logistic regression. In bivariable conditional fixed-effect logistic regression, receiving education intervention, place of delivery, pre-lacteal feeding, colostrum feeding and postnatal care were significantly associated with exclusive breast-feeding practices. In multivariable analysis, receiving education intervention and place of delivery were significantly associated with exclusive breast-feeding practices. Women who received education intervention group were 1·72 times more likely [AOR 1·72, 95 % CI (1·12, 2·64)] to practice exclusive breast-feeding than those who did not gain. Additionally, women who gave birth in health facilities were 2·36 times more likely [AOR 2·36, 95 % CI (1·28, 4·33)] to practice exclusive breast-feeding than those who gave birth at home (Table 6).

**Table 6.** Determinants of EBF among women in Hawela Tulla sub-city of Hawassa town, in 2020 (*n* 285)

Variables	Total	Exclusive breast-feeding		COR (95 % CI)	AOR (95 %)
		Yes	No		
Intervention					
Yes	143	106(74·1 %)	37(25·9 %)	1·700(1·122, 2·577)*	1·718(1·120, 2·635)**
No	142	86(60·6 %)	56(39·4 %)	1	1
Age					
15–19	23	12(52·2 %)	11(47·8 %)	0·632(0·320, 1·248)	0·970(0·413, 2·279)
20–24	105	69(65·7 %)	36(34·3 %)	0·621(0·317, 1·215)	1·216(0·438, 3·374)
25–29	119	81(68·1 %)	38(31·9 %)	0·509(0·216, 1·198)	0·649(0·155, 2·714)
≥30	38	30(78·9 %)	8(21·1 %)	1	1
Maternal educational status					
Cannot read and write	103	68(66·0 %)	35(34·0 %)	0·983(0·574, 1·683)	1·326(0·629, 2·797)
Can read and write	62	40(64·5 %)	22(35·5 %)	0·909(0·560, 1·475)	1·311(0·576, 2·984)
Primary (1–8)	96	66(68·8 %)	30(31·2 %)	0·648(0·273, 1·539)	1·120(0·347, 3·616)
Secondary and above	24	18(75·0 %)	6(25·0 %)	1	1
Partner's educational status					
Cannot read and write	76	46(60·5 %)	30(39·5 %)	0·853(0·457, 1·591)	0·773(0·350, 1·706)
Can read and write	45	29(62·2 %)	16(37·8 %)	0·840(0·510, 1·384)	0·685(0·300, 1·560)
Primary (1–8)	101	68(67·3 %)	33(32·7 %)	0·633(0·344, 1·166)	0·461(0·164, 1·298)
Secondary and above	63	49(77·8 %)	14(22·2 %)	1	1
Wealth index					
Poorest	22	11(50·0 %)	11(50·0 %)	0·672(0·342, 1·320)	1·150(0·509, 2·599)
Poorer	102	65(63·7 %)	37(36·3 %)	0·493(0·246, 0·988)	1·013(0·414, 2·479)
Middle	106	77(72·6 %)	29(27·4 %)	0·531(0·234, 1·205)	1·724(0·552, 5·384)
Richer	41	29(70·7 %)	12(29·3 %)	0·637(0·221, 1·835)	2·108(0·433, 10·264)
Richest	14	10(71·4 %)	4(28·6 %)	1	1
Parity					
Primipara	61	34(55·7 %)	27(44·3 %)	1	1
1–2	164	113(68·9 %)	51(31·1 %)	1·600(0·868, 2·948)	1·876(0·664, 5·297)
≥3	60	45(75·0 %)	15(25·0 %)	1·095(0·632, 1·899)	0·998(0·463, 2·148)
Number of ANC visits					
1–3	187	120(64·2 %)	67(35·8 %)	1	1
≥4	98	72(73·5 %)	26(26·5 %)	1·319(0·843, 2·064)	0·964(0·594, 1·566)
Place of delivery					
Health institution	198	151(76·3 %)	47(23·7 %)	2·381(1·585, 3·578)*	2·355(1·281, 4·328)**
Home	87	41(47·1 %)	46(52·9 %)	1	1
Delivery mode					
SVD	225	152(67·6 %)	73(32·4 %)	1	1
C/S	60	40(66·7 %)	20(33·3 %)	1·028(0·621, 1·702)	1·525(0·834, 2·789)
Sex of infant					
Male	138	97(70·3 %)	41(29·7 %)	1·112(0·740, 1·672)	1·240(0·809, 1·901)
Female	147	95(64·6 %)	52(35·4 %)	1	1
Pre-lacteal given					
No	220	159(72·3 %)	61(27·7 %)	1	1
Yes	65	33(50·8 %)	32(49·2 %)	0·438(0·286, 0·671)*	0·701(0·368, 1·335)
Colostrum given					
Yes	252	177(70·2 %)	75(29·8 %)	1	1
No	33	15(45·5 %)	18(54·5 %)	0·483(0·289, 0·809)*	0·812(0·401, 1·646)
Postnatal care					
Yes	228	165(72·4 %)	63(27·6 %)	2·155(1·394, 3·331)*	1·382(0·830, 2·303)
No	57	27(47·4 %)	30(52·6 %)	1	1

## Discussion

In the present study, women who received education intervention were more likely to practice early initiation of breast-feeding and exclusive breast-feeding than those who did not receive it. Maternal breast-feeding education, place of delivery, mode of delivery and prolateral feeding were determinants of early initiation of breast-feeding. Furthermore, maternal breast-feeding education and place of delivery were determinants of exclusive breast-feeding.

The present study revealed that the proportion of mothers who practiced early initiation of breast-feeding was significantly higher among women who received education intervention than those who did not receive (72·7 % *v.* 59·9 %). This result is consistent with a cluster randomised controlled trial done in Bangladesh in which significantly higher proportion of women in the intervention group practiced early initiation of breast-feeding compared to the control group (81·9 % *v*. 77·4 %)^(46)^. This result is also in agreement with the findings of studies done in Pakistan and India in which significantly higher proportion of women who received counselling intervention on breast-feeding-initiated breast-feeding earlier when compared to those who did not receive (96 % *v*. 84 % and 90·3 % *v.* 53·7 %, respectively)^(30)^. This could be explained by the education intervention might improve their knowledge about an advantage of early initiation of breast-feeding, and it might improve their motivation to initiate breast-feeding early.

Study participants who received education intervention were 1·55 times more likely to practice early initiation of breast-feeding than their counterparts were. This finding agrees with a study done in the Tayo District of Ethiopia which showed women who were given advice/information about timely initiation of breast-feeding were 3·71 times more likely to initiate breast-feeding early as compared to those who did not get^(47)^. A cluster randomised controlled trial study done in Bangladesh also showed that mothers in the control group were 2·53 times more likely to delay initiation of breast-feeding compared with mothers in the intervention group^(46)^. This might be due to increased awareness of women in the intervention group about the benefit's early initiation of breast-feeding. Additionally, the education given might make them have increased motivation to initiate breast-feeding earlier. On the contrary, the finding of the present study does not agree with a previously done quasi-experimental study in Hawassa town in which no statistically significant difference regarding early initiation of breast-feeding was noted between women in the intervention group compared with their counter group (77·7 % *v.* 69·9 %)^(15)^. This might be due to the basic water, sanitation and hygiene are among other strategies for diarrhoea prevention that are influenced by maternal age, educational status, occupation and household living conditions^(48)^.

Place of delivery was significantly associated with early initiation of breast-feeding. Women who gave birth in health facilities were 2·29 times more likely to initiate breast-feeding early than women compared to those who gave birth at home. This finding is in agreement with a study conducted in Bahir Dar city which showed women who gave birth at health institutions were 3·36 times more likely to initiate breast-feeding within 1 h of birth than those who gave birth at home^(49)^. A meta-analysis study was done in Ethiopia also indicated that mothers who gave birth at the health institution were 2·11 times more likely to initiate breast-feeding within 1 h of birth as compared to mothers who gave birth at home^(12)^. The finding of the present study was also similar to a study done in rural Tanzania which showed women who give birth in health facilities were almost twice more likely to initiate breast-feeding early compared with those who gave birth at home^(50)^. This might be because mothers who gave birth at health institutions have a good opportunity to receive counselling and assistance from health professionals regarding the early initiation of breast-feeding. Another explanation could be giving birth in health facilities gives women the confidence to challenge perceived cultural practices, myths and belief systems held towards breast-feeding in the community^(46)^.

In the present study, mode of delivery was also significantly associated with early initiation of breast-feeding. Women who gave birth vaginally were 2·85 times more likely to initiate breast-feeding early as compared with those who gave birth by caesarean section. This finding had agreement with the study conducted in south Gondar which indicated mothers who delivered vaginally were four times more likely to initiate breast-feeding early than their respective counterparts^(18)^. Additionally, the present study agrees with a study done in Nigeria which indicated the odds of EIBF were three times higher for mothers who had vaginal delivery as compared to mothers who had caesarean section^(51)^. This could be explained by the morbidity associated with exhaustion from a difficult labour, the effect of anaesthesia, the emotional adjustment to the fact that the mother was unable to deliver normally and severe abdominal pain from the wound^(51,52)^. Another explanation could be due to the fact that infants born from women who have given birth by caesarean section remain in the hands of the attendants until their mothers recover from anaesthesia. These might cause a long delay in making the first contact with mothers with their infants, and mothers might also find it difficult to achieve comfortable breast-feeding positions^(53)^.

Furthermore, mothers who gave pre-lacteal feeds to infants within 3 d of life were 53 % less likely to initiate breast-feeding within 1 h after birth. The finding had an agreement with a study done in a rural part of West Ethiopia which showed infants who were provided with pre-lacteal feeds were 70 % less likely to initiate breast-feeding early^(20)^. The findings of a study done in Saudi Arabia had also indicated similar results in which women who gave pre-lacteal feeds were 70 % less likely to initiate breast-feeding early^(54)^. Pre-lacteal feeding results in the baby receiving insufficient breast milk possibly leading to lactation failure and weakening the suckling stimulus^(20)^. Additionally, when the infants were given pre-lacteal feeds, breast-feeding initiation will be delayed.

Exclusive breast-feeding practice was also significantly higher among women who received education intervention than those who did not receive it (74·1 % *v*. 60·6 %). This result is similar to a previously done study in Arba Minch Zuria Woreda which revealed a significantly higher proportion of women who received health education about breast-feeding practiced exclusive breast-feeding than those who did not receive the education (76·40 % *v*. 43·60 %)^(55)^. Another study done in Pakistan also showed that the practice of exclusive breast-feeding was statistically higher among mothers who received counselling on breast-feeding than those who did not receive (68 % *v.* 16 %)^(29)^. Similarly, another interventional study done in India found a significant difference in the practice of exclusive breast-feeding between women in the intervention and control groups (91 % *v.* 43 %)^(56)^. This could be due to women who have received education on breast-feeding were better to understand the rationale behind exclusive breast-feeding.

Study participants who received education intervention were 1·72 times more likely to practice exclusive breast-feeding than those women who did not receive education intervention. This finding is consistent with a study done in Bahir Dar city, Northwest Ethiopia which indicated women who received infant feeding counselling/advice were 5·20 times more likely to exclusively breast-feed their infants^(57)^. Another study done in Bangladesh showed that mothers in the intervention group were 5·10 times more likely to practice exclusive breast-feeding compared with mothers in the control group^(46)^. Similarly, a systematic review and meta-analysis study conducted in Iran found that women who received breast-feeding education were 1·13 times more likely to practice exclusive breast-feeding than those who did not received education on breast-feeding^(58)^. This could be because the education given to women have impacted on the three dimensions of knowledge, attitudes and behaviour of the mothers and encouraged them to practice exclusive breast-feeding^(59)^.

Women who gave birth in health facilities were 2·36 times more likely to practice exclusive breast-feeding than those who gave birth at home. This result agrees with a systematic review and meta-analysis study done in Ethiopia which found mothers who gave birth in a health institution were 2·2 times more likely practicing EBF compared to mothers who gave birth at home^(13)^. Similarly, studies done in Bahir Dar and Debre Tabor Town showed that women who delivered in a health facility were 3·02 and 3·8 times more likely to practice exclusive breast-feeding, respectively, as compared to those who delivered at home^(57,60)^. This could be due to the fact that mothers who give birth in health institutions have more opportunities to obtain counselling and support by health professionals. On the other hand, this finding contradicts with a study reported from the rural area of Sorrow District, Southern Ethiopia in which place of delivery is not significantly associated with exclusive breast-feeding^(61)^. This could be explained by socioeconomic and cultural variation across the study participants.

## Strengths and limitations

The cluster randomised controlled study might be the study strength that facilitates blinding to give an equal chance of participation and minimises bias, particularly selection bias and confounding. The powers and precision of a cluster randomised trial is lower than an individually randomised trial. Other issues to consider are imbalance between study arms, and generalizability might be a possible limitation.

## Conclusion

As conclusion, breast-feeding education given to women was effective in significantly improving EIBF and EBF practices among women in the intervention group. Among the different factors included in the study, maternal breast-feeding education, institutional delivery, vaginal mode of delivery and pre-lacteal feeding were determinants of early initiation of breast-feeding. Furthermore, maternal breast-feeding education and institutional delivery were determinants of exclusive breast-feeding.

## Recommendations

### To HEWs and other healthcare providers

Providing sustainable education about benefits of optimal breast-feeding practices with particular attention to EIBF and EBF both at the community and health facility level integrating services with essential nutrition action contact points.

Strengthening breast-feeding education discourages pre-lacteal feeding. Strengthening the efforts to encourage pregnant women to give birth in health institutions. Particular attention should be given to women who gave birth by caesarean section to breast-feed their infants.

### To researchers

It is better if qualitative and quantitative triangulated longitudinal studies involving with a large sample size are conducted to further explore the determinants of EIBF and EBF.
